# Lifestyle risk indices in adolescence and their relationships to adolescent disease burden: findings from an Australian national survey

**DOI:** 10.1186/s12889-019-6396-y

**Published:** 2019-01-14

**Authors:** Louise Mewton, Katrina Champion, Frances Kay-Lambkin, Matthew Sunderland, Louise Thornton, Maree Teesson

**Affiliations:** Centre of Research Excellence in Mental Health and Substance Use, National Drug and Alcohol Research Centre, University of New South Wales, 22-32 King St, Randwick, NSW 2032 Australia

**Keywords:** Adolescence, Lifestyle risk factors, Burden of disease

## Abstract

**Background:**

The current study investigates the extent to which an adolescent-specific lifestyle risk factor index predicts indicators of the leading causes of adolescent morbidity and mortality.

**Methods:**

Data came from 13 to 17 year-old respondents from the 2013–2014 nationally representative Australian Child and Adolescent Survey of Mental Health and Wellbeing (*n* = 2314). Indicators of adolescent disease burden included Major Depressive Disorder, psychological distress, self-harm and suicide attempt. Risk factors included risky alcohol use, drug use, unprotected sex, smoking, BMI and sleep duration. The extent to which these risk factors co-occurred were investigated using tetrachoric correlations. Several risk indices were then constructed based on these risk factors. Receiver Operating Characteristic curves determined the precision with which these indices predicted the leading causes of adolescent disease burden.

**Results:**

Risky alcohol use, drug use, smoking, unprotected sex, and sleep were all highly clustered lifestyle risk factors, whereas BMI was not. A risk index comprising risky alcohol use, drug use, unprotected sex and sleep duration predicted the disease burden outcomes with the greatest precision. 31.9% of the sample reported one or more of these behaviours.

**Conclusions:**

This lifestyle risk factor index represents a useful summary metric in the context of adolescent health promotion and non-communicable disease prevention. Lifestyle risk factors were found to cluster in adolescence, supporting the implementation of multiple health behaviour change interventions.

## Background

Whilst adolescence and young adulthood is often thought of as a period of relative good health, burden of disease studies indicate that10–24 year olds remain at substantial risk of morbidity and mortality [1]. Burden of disease in this age group is not driven by physical illness, but by mental illness, self-harm and suicide. Meanwhile, the leading risk factors contributing to this burden of disease in young people include risky alcohol use, illicit drug use and unprotected sex [2, 3]. These risk factors are not only associated with poor outcomes for youth in the short-term, such as obesity and mental health problems, but are also associated with morbidity and mortality experienced later in life [4–7]. Evidence has consistently shown that these risk behaviours are linked to the development of later chronic non-communicable conditions, such as heart disease, cancer and Type II diabetes, which cause considerable disease burden amongst older adults [8–10]. Experts have therefore suggested that chronic health disease risk factor management should begin in adolescence, as evidence is accumulating on the benefits of early intervention for future health gains [11]. Identifying clusters of potentially modifiable risk behaviours associated with morbidity and mortality amongst adolescents, and risk for later chronic disease in adulthood, would ideally guide the development of preventive interventions designed to reduce the burden of disease across the lifespan.

Previous research has linked certain clusters of risk factors with indicators of mental health in young people. For example, the combination of concurrent risky alcohol use, drug use and smoking is related to increased depressive symptom severity in young people aged 15–30 years [12], as has the clustering of physical inactivity, smoking, low-quality diet, and abnormal (high or low) body mass index (BMI) [4]. The cluster of smoking, alcohol misuse, marijuana use, sexual activity, violence, and suicidality has also predicted a range of psychopathology in children and adolescents aged 9–17 years, including mood, anxiety and disruptive disorders [13]. In another study, risky alcohol use, illicit drug use, smoking, sleep deprivation, overweight/underweight, sedentary behaviour, high media use, and truancy were related to a range of poor mental health outcomes, including depression, anxiety and suicidality amongst adolescents (mean age 14.9 years) [14]. Recent research has therefore recognised the importance of lifestyle risk factors in the determination of adolescent mental health. In the adult literature, the importance of these risk factors has led to the development of lifestyle risk indices comprised of factors related to disease burden in adults [7, 15]. To our knowledge, a similar index of adolescent-specific risk factors for burden of disease has not been constructed. It is envisaged that this lifestyle risk factor index could be used to easily identify adolescents at risk for chronic diseases of both adolescence and adulthood in a rigorous and standardised way. Those identified as at risk could then be targeted by healthy lifestyle interventions designed to reduce the burden of disease across the lifespan.

Using a large nationally representative sample of Australian adolescents, the current study will construct a lifestyle risk index comprising the leading risk factors for adolescent burden of disease (i.e., risky alcohol use, drug use and unprotected sex) [3]. The extent to which this index predicts indicators of the leading causes of adolescent morbidity and mortality (i.e., depression, psychological distress, self-harm and suicide attempt) will also be investigated. The study will also investigate whether the addition of conventional (i.e., smoking and BMI) and emerging (i.e., sleep duration) risk factors associated with disease burden in adulthood [7, 14, 16, 17] improves the measurement properties of this index.

## Methods

### Sample

The 2013–2014 s Australian Child and Adolescent Survey of Mental Health and Wellbeing was based on a stratified, multistage area probability sample of households where there was at least one child aged 4–17 years. The survey represents the most up-to-date, detailed snapshot of mental health and wellbeing in adolescents in Australia. In total 6310 parents and carers of eligible households participated in the survey, representing an overall response rate of 55%. In addition, 2967 (89%) of young people aged 11–17 years, for whom their parents or carers had given written consent, also gave written consent to complete a youth self-report questionnaire. Comparison with 2011 Australian Census data indicated that the sample was broadly representative of the Australian population in terms of demographic characteristics [18]. The survey data were weighted to represent the Australian population of children and adolescents aged 4–17 years based on 2013 data from the Australian Bureau of Statistics. The current study uses data from youth aged 13–17 years (*n* = 2314). Those aged 11–12 did not provide data on the relevant risk behaviours and were therefore excluded from the current study. The survey received ethical approval from the Australian Government Department of Health. The survey methods have been discussed in more detail elsewhere [18] and full questionnaires used in the survey can be accessed here: https://youngmindsmatter.telethonkids.org.au/for-researchers/.

## Measures

### Key variables accounting for morbidity/mortality in adolescence

The 2013–2014 s Australian Child and Adolescent Survey of Mental Health and Wellbeing included a self-report module based on the Diagnostic Interview Schedule for Children (DISC-IV) which was used to provide a 12-month diagnosis of major depressive disorder (MDD) [19]. The DISC-IV is designed for use in children and adolescents aged 6–17 years and has been shown to have acceptable test-retest reliability in community samples [19]. Questions from the Youth Risk Behavior Surveillance System (YRBSS) were also included in the survey and were used to provide data for the suicide attempt and self-harm outcomes [20]. Participants were asked whether they had attempted suicide in the past 12 months (suicide attempt) and whether they had deliberately harmed or injured themselves without intending to end their own life during the past 12 months (self-harm). Psychological distress in the past 4 weeks was measured by the 10-item Kessler Psychological Distress scale (K10) [21], which has been used previously in samples of adolescents in the Australian general population [22], and demonstrated excellent reliability in the current sample (Cronbach’s α = 0.91). Those reporting severe psychological distress (scores 21 and above) were compared with those with scores below this standard cut-off [23]. The dichotomised version of the K10 has been used extensively, and has been shown to predict serious mental illness in both adolescents and adults with good accuracy [22, 24, 25].

### Risk factors

Self-reported risky alcohol use, drug use, smoking, and unprotected sex were collected as part of the YRBSS. To evaluate an index based on these risk factors, each was coded as 0 (not at risk) or 1 (at risk). Alcohol risk was defined as consuming four or more standard drinks on a single occasion in the past 30 days, based on the Australian National Health and Medical Research Council’s definition of single occasion risky drinking [26]. Respondents were asked to select which drugs they had used in the past 30 days from a list including cannabis, meth/amphetamine, cocaine, ecstasy and prescription drugs for non-medical or non-prescribed purposes. Drug risk was defined as the use of any of these illicit drugs in the past 30 days. Those who had smoked cigarettes in the past 30 days were considered at smoking risk. Respondents were asked whether they used any form of protection to prevent pregnancy or sexually transmitted infections. Sexual risk was defined as those who reported using no form of protection the last time they had sexual intercourse. Youth were also asked to self-report their current height and weight [used to derive BMI (height (m)/weight^2^ (kg)] and the average amount of sleep each week night and each weekend night (hours). Those with a BMI less than 18.5 or higher than 30 were considered at weight risk, according to standard guidelines of underweight and obesity from the Australian Government Department of Health [27]. Both ends of the BMI spectrum were considered given evidence suggesting that the relationship between mental health and BMI is U-shaped, with those who are both underweight and overweight at higher risk for mental health problems such as depression [28]. In addition, both weight gain and weight loss are considered symptoms of major depression [29]. Finally those who slept less than 7 hours and more than 11 hours per night on average were considered at sleep risk, according to Australian sleep guidelines for adolescents [30]. Sleep problems, which include both hypersomnia and insomnia, are associated with mental health problems, and are both considered symptoms of major depression [29].

We constructed a basic risk index, which consisted of risky alcohol use, drug use and unprotected sex, the leading risk factor associated with adolescent disease burden [2, 3]. The basic risk index scores ranged from 0 to 3. Three other specific risk indices were then created, comprising the basic index + smoking, the basic index + BMI and the basic index + sleep. Each of the specific risk index scores ranged from 0 to 4. The basic index + smoking + BMI, the basic index + smoking + sleep, and the basic index + BMI + sleep were also constructed (scores ranging from 0 to 5). Finally, an index consisting of all six risk factors (risky alcohol use, drug use, unprotected sex, smoking, BMI and sleep) was constructed (ranging from 0 to 6).

Table 1 includes a description of each of the risk factors, as well as a summary of how each of the risk indices were composed.

**Table 1:** Lifestyle factors, risk scoring methods and risk indices derived from the 2013–2014 Australian Child and Adolescent Survey of Mental Health and Wellbeing (*n* = 2314)

		Risk indices							
Lifestyle factor	Risk Scoring Method	Basic risk	Basic risk + BMI	Basic risk + Sleep	Basic risk + Smoking	Basic risk + BMI + Sleep	Basic risk + BMI + Smoking	Basic risk + Sleep + Smoking	Basic risk + BMI + Sleep + Smoking
Alcohol use	*1 = 4 or more drinks on a single occasion in past month*	✓	✓	✓	✓	✓	✓	✓	✓
Illicit drug use	*1 = past 30-day illicit drug use*	✓	✓	✓	✓	✓	✓	✓	✓
Unprotected sex	*1 = no protection used against pregnancy or sexually transmitted diseases the last time respondent had sexual intercourse*	✓	✓	✓	✓	✓	✓	✓	✓
BMI	*1 = Body mass index < 18.5 or > 30*		✓			✓	✓		✓
Sleep	*1 = < 7 h or > 11 h/day*			✓		✓		✓	✓
Smoking	*1 = past 30-day cigarette smoking*				✓		✓	✓	✓

### Statistical analyses

All analyses were weighted to account for the complex survey design and were conducted using SAS 9.4. The prevalence of each risk factor and the four leading causes of disease burden in adolescence (self-reported MDD, suicide attempt, self-harm, severe psychological distress) were first analysed by key demographic variables: age, sex, rurality (classified as greater capital cities or rest of state, as per the Australian Bureau of Statistics’ Australian Statistical Geography Standard), country of birth and current education status (whether or not the participant currently goes to school, as reported by the parent). The extent to which the risk factors co-occurred was also investigated using tetrachroic correlations. Preliminary analyses focused on whether the basic risk index (alcohol use, drug use and unprotected sex) predicted the four disease burden outcomes with greater precision than any single risk factor, or combination of two risk factors, that comprised this index. The predictive utility of the basic risk index was then compared with the seven other indices constructed as described above and in Table 1. The predictive utility of these various combinations of risk factors was assessed using receiver operating characteristic (ROC) curves created with logistic regressions that controlled for demographic characteristics (age, sex, country of birth and education status) which were likely to be related to both the risk factors and indicators of burden of disease in young people.. The area under the curve (AUC) was calculated to describe how well each index classified the four causes of disease burden in adolescence, and to compare these indices in terms of predictive value. An AUC of 0.5 indicates that an index is no better than chance at predicting risk, whilst an AUC of 1.0 indicates that an index predicts risk perfectly. Areas under the curve for each index were compared with the basic risk index (reference index) using the ROCCONTRAST statement in SAS 9.4 which implements a nonparametric approach as recommended by methodological experts [31].

An optimal at-risk threshold (or cut-off) was then established for the index that predicted the four outcome variables with the highest precision. This optimal threshold was selected by comparing AUCs characterising the relationship between all possible thresholds on the best performing index and the four disease burden outcome variables of interest. A threshold of one risk factor was first imposed on the best performing index, and AUCs were constructed describing the precision with which one risk factor predicted each of the disease burden outcome variables. A threshold of two risk factors was then imposed and AUCs were again constructed describing the precision with which a total of two risk factors predicted each of the disease burden outcomes variables. This procedure was then replicated for each possible threshold on the best performing risk index. Basic demographic characteristics of those identified as at-risk according to this optimal threshold were then described using univariate and multivariate logistic regression models.

## Results

Overall, 48.7% of the sample were female, the mean age was 15.0, 63.5% of the sample were from metropolitan areas, 85.8% were Australian born and 93.0% of the sample were currently attending school. Descriptive statistics by causes of disease burden outcomes and major risk behaviours are presented in Table 2. Based on tetrachoric correlations (Table 3), risky alcohol use, drug use, smoking, unprotected sex, and sleep were all significantly and highly clustered risk factors. BMI did not appear to cluster with these other factors.

**Table 2:** Prevalence of lifestyle risk factors by demographic characteristics in the 2013–2014 Australian Child and Adolescent Survey of Mental Health and Wellbeing (n = 2314)

	Major depression % (SE)	Self-harm % (SE)	Suicide attempt % (SE)	Severe psychological distress % (SE)	Risky alcohol use % (SE)	Illicit drug use % (SE)	Unprotected sex % (SE)	Sleep % (SE)	Smoking % (SE)	BMI% (SE)
Sex										
Male	5.4 (0.6)	4.4 (0.6)	1.3 (0.3)	3.7 (0.6)	12.6 (1.0)	5.6 (0.7)	1.0 (0.3)	18.7 (1.2)	6.2 (0.8)	27.1 (1.5)
Female	15.5 (1.1)	13.7 (1.0)	4.0 (0.6)	9.2 (0.9)	12.5 (1.0)	5.5 (0.7)	2.1 (0.5)	21.9 (1.3)	8.2 (0.8)	29.2 (1.6)
Age										
13	4.2 (1.2)	5.3 (1.3)	1.0 (0.6)	2.1 (0.8)	1.0 (0.7)	1.9 (0.9)	0.5 (0.5)	15.5 (2.2)	1.2 (0.7)	42.5 (3.0)
14	7.3 (1.4)	7.9 (1.5)	2.3 (0.9)	7.4 (1.5)	4.2 (1.2)	2.7 (0.8)	0.5 (0.5)	17.2 (2.2)	4.8 (0.3)	31.9 (2.6)
15	10.7 (1.8)	8.3 (1.6)	2.2 (0.8)	5.7 (1.4)	8.1 (1.6)	5.0 (1.3)	1.6 (0.7)	18.6 (2.4)	5.8 (1.4)	26.7 (2.7)
16	15.6 (1.4)	12.1 (1.3)	3.9 (0.7)	8.0 (1.0)	20.9 (1.6)	10.4 (1.2)	2.4 (0.6)	24.2 (1.6)	11.4 (1.2)	20.6 (1.6)
17	13.8 (1.4)	11.2 (1.3)	3.7 (0.7)	8.7 (1.1)	27.8 (1.9)	7.5 (1.1)	2.7 (0.7)	25.6 (1.8)	12.4 (1.6)	20.7 (1.7)
Area										
City	10.5 (0.8)	8.7 (0.8)	2.4 (0.4)	6.6 (0.7)	11.7 (0.9)	5.7 (0.6)	1.5 (0.3)	20.4 (1.2)	6.6 (0.6)	28.4 (1.3)
Rural	10.0 (1.1)	9.4 (1.1)	3.1 (0.7)	6.0 (0.9)	14.0 (1.2)	5.2 (0.8)	1.7 (0.5)	20.0 (1.6)	8.2 (1.2)	27.8 (2.1)
Country of birth										
Australia	10.8 (0.7)	9.2 (0.7)	3.0 (0.4)	6.7 (0.6)	13.5 (0.8)	6.0 (0.6)	1.7 (0.3)	19.7 (1.0)	7.7 (0.6)	27.7 (1.2)
Overseas	7.8 (1.3)	7.8 (1.5)	0.6 (0.4)	4.7 (2.5)	6.9 (1.3)	2.8 (0.9)	0.8 (0.4)	23.8 (2.5)	4.2 (1.1)	30.5 (2.9)
Education										
Not attending school	19.9 (2.7)	15.5 (2.5)	4.8 (1.6)	9.7 (2.1)	37.9 (3.3)	16.6 (2.6)	8.1 (1.9)	28.4 (3.0)	28.5 (3.4)	18.6 (2.8)
Attending school	9.6 (0.6)	8.5 (0.6)	2.5 (0.3)	6.1 (0.6)	10.6 (0.7)	4.7 (0.5)	1.1 (0.3)	19.6 (1.0)	5.6 (0.5)	28.9 (26.5)
Total	10.4 (0.6)	9.0 (0.86)	2.6 (0.3)	6.4 (0.5)	12.5 (0.7)	5.5 (0.5)	1.6 (0.3)	20.3 (1.0)	7.2 (0.6)	28.1 (1.1)

NB: Risky alcohol use defined as 4 or more standard drinks in past 30 days; illicit drug use risk defined as any drug use in past 30 days; unprotected sex risk defined as no protection against pregnancy of sexually transmitted diseases the last time the respondent had sexual intercourse; BMI (body mass index) risk defined as less than 18.5 or greater than 30; sleep risk defined as less than 7 hours per day or more than 11 hours per day on average; smoking risk defined as cigarette smoking in the past 30 days

**Table 3:** Tetrachoric correlations between lifestyle risk factors included in the 2013–2014 Australian Child and Adolescent Survey of Mental Health and Wellbeing (*n* = 2314)

	Risky Alcohol Use	Illicit Drug Use	Unprotected Sex	BMI	Sleep	Smoking
Alcohol use	1.00	0.69^b^	0.25^a^	−0.14^a^	0.13^a^	0.65^b^
Illicit drug use		1.00	0.47^b^	0.01	0.27^b^	0.77^b^
Unprotected sex			1.00	0.01	0.08	0.51^b^
BMI				1.00	−0.04	0.05
Sleep					1.00	0.30^b^
Smoking						1.00

^a^*p* < 0.01; ^b^ *p* < 0.0001

NB: Risky alcohol use defined as 4 or more standard drinks in past 30 days; illicit drug use risk defined as any drug use in past 30 days; unprotected sex risk defined as no protection against pregnancy of sexually transmitted diseases the last time the respondent had sexual intercourse; BMI (body mass index) risk defined as less than 18.5 or greater than 30; sleep risk defined as less than 7 hours per day or more than 11 hours per day on average; smoking risk defined as cigarette smoking in the past 30 days

Preliminary analyses focused on validating the basic risk index comprising alcohol use, drug use and unprotected sex. The basic risk index was found to predict the four disease burden outcomes with fair to good precision (Table 4), and with more accuracy than any of the composite risk factors either alone (i.e., alcohol use, drug use or unprotected sex) or in combination (i.e., alcohol use + drug use, alcohol use + unprotected sex or drug use + unprotected sex) (results available on request). Table 4 presents the AUCs for the basic risk index, as well as comparisons between this index and the AUCs for each of the calculated risk indices, in terms of their ability to predict the four disease burden outcomes. When comparing the different indices, the basic risk + sleep + smoking index predicted the disease burden outcomes with the greatest precision, and with greater precision than the basic risk index alone (all contrast *p*s < .01). The basic risk + sleep + smoking index was therefore selected as the most precise risk index predicting adolescent disease burden. Based on the basic risk + sleep + smoking index, 68.1% of the sample reported no risk behaviours, 22.7% reported one risk behaviour, 4.8% reported two risk behaviours, 3.2% reported three risk behaviours, 1.1% reported four risk behaviours, and 0.1% reported all five risk behaviours.

**Table 4:** Area under the curves for each lifestyle risk index and causes of adolescent disease burden in the 2013–2014 Australian Child and Adolescent Survey of Mental Health and Wellbeing (*n* = 2314)

	Risk indices							
	Basic risk AUC (95% CI)	Basic risk + BMI AUC (95% CI)	Basic risk + Sleep AUC (95% CI)	Basic risk + Smoking AUC (95% CI)	Basic risk + BMI + Sleep AUC (95% CI)	Basic risk + BMI + Smoking AUC (95% CI)	Basic risk + Sleep + Smoking AUC (95% CI)	Basic risk + BMI + Sleep + Smoking AUC (95% CI)
Major depression	0.706 (0.671–0.742)	0.702 (0.667–0.736)	0.753 (0.718–0.787) ^c^	0.713 (0.678–0.748)	0.739 (0.705–0.773) ^c^	0.709 (0.675–0.743)	0.757 (0.723–0.791) ^c^	0.743 (0.709–0.776) ^c^
Self-harm	0.717 (0.684–0.751)	0.710 (0.677–0.743)	0.743 (0.711–0.776) ^b^	0.723 (0.690–0.757)	0.729 (0.697–0.762)	0.717 (0.684–0.750)	0.747 (0.715–0.780) ^b^	0.736 (0.703–0.768) ^a^
Suicide attempt	0.765 (0.708–0.823)	0.766 (0.708–0.824)	0.826 (0.777–0.875) ^c^	0.791 (0.736–0.847) ^a^	0.820 (0.775–0.864) ^c^	0.798 (0.742–0.855) ^a^	0.838 (0.793–0.884) ^c^	0.836 (0.793–0.880) ^c^
Severe psychological distress	0.701 (0.655–0.747)	0.693 (0.649–0.738)	0.759 (0.717–0.802) ^c^	0.709 (0.663–0.754)	0.737 (0.694–0.779) ^b^	0.701 (0.656–0.747)	0.768 (0.727–0.810) ^c^	0.743 (0.702–0.785) ^b^

^a^contrast with basic risk index *p* < 0.05; ^b^ contrast with basic risk index *p* < 0.01; ^c^ contrast with basic risk index *p* < 0.001

NB: Risky alcohol use defined as 4 or more standard drinks in past 30 days; illicit drug use risk defined as any drug use in past 30 days; unprotected sex risk defined as no protection against pregnancy of sexually transmitted diseases the last time the respondent had sexual intercourse; BMI (body mass index) risk defined as less than 18.5 or greater than 30; sleep risk defined as less than 7 hours per day or more than 11 hours per day on average; smoking risk defined as cigarette smoking in the past 30 days

A threshold of one or more risk behaviours on the basic risk index + sleep + smoking index was the best predictor of depression [AUC = 0.747 (95% CI: 0.715, 0.779); sensitivity = 0.667 (95% CI: 0.605–0.730); specificity = 0.718 (95% CI: 0.696–0.741)], self-harm [AUC = 0.731 (95% CI: 0.700, 0.762); sensitivity = 0.637 (95% CI: 0.569–0.705); specificity = 0.710 (95% CI: 0.688–0.732)] and severe psychological distress [AUC = 0.753 (95% CI: 0.714, 0.793); sensitivity = 0.705 (95% CI: 0.626–0.784); specificity = 0.705 (95% CI: 0.683–0.727)]. A threshold of two risk behaviours was a marginally better predictor for suicide attempt [one behaviour AUC = 0.797 (95% CI: 0.761, 0.832); sensitivity = 0.827 (95% CI: 0.726–0.928); specificity = 0.692 (95% CI: 0.670–0.715); two behaviour AUC = 0.806 (95% CI: 0.754, 0.858); sensitivity = 0.626 (95% CI: 0.497–0.756); specificity = 0.919 (95% CI: 0.907–0.931)], but this difference was not statistically significant (*p* = 0.689). A threshold of one or more risk behaviours on the basic risk index + sleep + smoking was therefore selected as the optimal cut-off in terms of predicting each the four causes of disease burden in adolescence. Overall, 31.9% of the sample scored above this threshold.

Table 5 displays the demographic characteristics of adolescents who were classified as ‘at risk’ according to the one or more threshold on the basic risk + sleep + smoking index. The odds of being classified as ‘at risk’ increased with age, and were greater amongst those not attending school.

**Table 5:** Demographic characteristics of at risk group (i.e., those reporting one or more behaviours on the basic risk + sleep + smoking index) in the 2013–2014 Australian Child and Adolescent Survey of Mental Health and Wellbeing (*n* = 2314)

	% (SE)	Unadjusted OR (95% CI)	Adjusted OR (95% CI)
Sex			
Male	30.4 (1.5)	1.17 (0.98–1.40)	0.87 (0.72–1.06)
Female	33.9 (1.5)	[ref]	[ref]
Age			
13	16.7 (2.4)	[ref]	[ref]
14	22.4 (2.4)	1.43 (0.93–2.21)	1.42 (0.92–2.19)
15	26.5 (2.6)	1.79 (1.126–2.78)^b^	1.75 (1.13–2.71)^a^
16	43.1 (1.9)	3.78 (2.62–5.45)^c^	3.55 (2.46–5.14)^c^
17	51.0 (2.1)	65.20 (3.60–7.50)^c^	4.05 (2.77–5.92)^c^
Area			
City	31.2 (1.4)	[ref]	[ref]
Rural	33.6 (1.9)	1.12 (0.91–1.37)	1.14 (0.92–1.42)
Country of birth			
Australia	32.3 (1.2)	1.06 (0.81–1.39)	1.05 (0.79–1.40)
Overseas	31.0 (2.7)	[ref]	[ref]
Education			
Not attending school	66.5 (3.3)	4.74 (3.51–6.41)^c^	2.67 (1.98–3.62)^c^
Attending school	29.5 (1.1)	[ref]	[ref]
Total	31.9% (1.1)	–	–

^a^*p* < 0.05; ^b^ *p* < 0.01; ^c^ p < 0.001

NB: Risky alcohol use defined as 4 or more standard drinks in past 30 days; illicit drug use risk defined as any drug use in past 30 days; unprotected sex risk defined as no protection against pregnancy of sexually transmitted diseases the last time the respondent had sexual intercourse; BMI (body mass index) risk defined as less than 18.5 or greater than 30; sleep risk defined as less than 7 hours per day or more than 11 hours per day on average; smoking risk defined as cigarette smoking in the past 30 days

## Discussion

In a large, nationally representative sample of Australian adolescents, this study investigated the clustering of lifestyle risk factors, and the extent to which these clusters predicted the key causes of excessive adolescent burden of disease. A lifestyle risk index comprised of risky alcohol use, drug use, unprotected sex, sleep duration and smoking predicted each of the disease burden outcomes with the best precision. When an empirically-derived threshold of one or more risk behaviours was imposed on this index, the prediction of these health outcomes ranged from fair to good. Such a high level of predictive precision was surprising, especially given the complexity of the disease burden outcomes investigated. These results suggest that this lifestyle risk index represents a useful summary metric in the context of health promotion and non-communicable disease prevention. Major depression is predicted to become the largest contributor to burden of disease by 2030 [32], and is associated with other non-communicable diseases that contribute excessively to the burden of disease in adults, such as heart disease, cancer and Type II diabetes [33]. Prevention programs targeting these novel and potentially modifiable lifestyle risk factors in adolescence may therefore be instrumental in reducing the considerable burden of disease associated with poor mental health across the lifespan. The current study proposes a lifestyle risk factor index that could be used in these prevention programs to identify those adolescents at risk for poor mental health outcomes and who my benefit from a healthy lifestyles intervention.

Of note, this study identified sleep as an important risk factor in the prediction of adolescent disease burden. This is consistent with similar studies of the adult population [7]. Recent “calls to action” have focused on the importance of sleep, particularly given its relationship with risk behaviours associated with weight gain [17]. Longitudinal studies have also found that poor sleep quality in early adolescence is associated with earlier onset of alcohol and cannabis use [16]. There is consistent evidence to therefore suggest that sleep is an important risk factor contributing to disease burden, and should be a target for future health promotion activities.

These findings have implications for the types of lifestyle factors that may be targeted by multiple health behaviour change interventions focusing on adolescents. Some of the lifestyle factors that form the risk index were adolescent-specific contributors to burden of disease (i.e., unprotected sex and drug use) [2, 3]. Risky alcohol use and sleep duration, however, have also been identified as important predictors of adult health outcomes [7, 8]. These findings suggest that focusing on these latter risk factors may be useful for addressing concurrent adolescent health as well as future morbidity and mortality associated with chronic diseases of adulthood. Thus, these lifestyle risk factors may therefore be particularly important to target in adolescent preventive interventions given their potential for both short- and long-term impact.

Consistent with previous research [4, 13, 14, 34–40], this study indicated that lifestyle risk factors tend to cluster together in adolescence. This clustering may occur through direct causation (i.e., unprotected sex as a result of alcohol or drug use) or shared aetiologies (i.e., social disadvantage). Given that these lifestyle risk factors are highly clustered, modifying one factor may also lead to changes in another. Historically, interventions aimed at preventing lifestyle risk factors have targeted a single factor [41]. However, the clustering of lifestyle risk factors suggests that multiple risk factors should be targeted together, rather than in isolation. Interventions designed specifically to target multiple risk factors are less resource intensive and more cost-effective than single factor interventions [41]. There is a growing literature on multiple health behaviour change interventions among adolescents and adults, but many of these are limited to small number of risk factors, and few include emerging risk factors, such as sleep duration which was identified as important in the current study [38, 41–45]. Future research should focus on developing and evaluating multiple health behaviour change interventions for adolescents and young adults, perhaps including the risk factors identified by the current study.

The current study also provides evidence as to the setting and timing of multiple health behaviour change interventions for adolescents. Youth outside of school settings are much more likely to be at risk in terms of the lifestyle factors included in this study. Whilst previous research indicates that school may be the best place for implementing “universal” prevention strategies [46], “selected” or “indicated” risk factor prevention could be considered as a means of targeting adolescents who are no longer attending school. Delivering interventions to high risk adolescents outside of school settings is a current challenge that should be prioritised. In the current study, the prevalence of these lifestyle risk factors increased with age, with a particularly sharp increase between the ages of 15 and 16 years. Although chronic disease prevention is advocated across the lifespan, the present findings suggest than an optimal time to deliver preventive interventions is in early adolescence, before the age 15, when these lifestyle risk factors are more likely to be initiated and before they become entrenched.

The findings from this study need to be considered within the context of its strengths and limitations. This study included data from a large, nationally representative sample of adolescents from the general population. In addition, health outcomes related to the leading global burdens of disease in adolescents were measured. Depression was assessed using a self-administered structured diagnostic questionnaire, and other health outcomes were assessed using standardised measures. Limitations of this study include its cross-sectional design. Longitudinal studies are needed to explore how these lifestyle risk factors co-vary over time to predict morbidity and mortality in adolescence through to adulthood. Risk factors and health outcomes were all self-reported, with the possibility of over- or under-reporting by participants especially with the querying of sensitive topics. Future research should also attempt to use objective measures to validate self-report of physical activity, sedentary behaviour and sleep patterns and sleep duration. There is an absence of energy balance risk behaviours in our risk index, and we included BMI as a proxy for these behaviours. Physical inactivity, diet and sedentary behaviour have been found to co-occur in adolescents [47] and to predict poor outcomes in adolescence [17] and the incidence of later cardiovascular disease, cancers and diabetes [48]. These measures were not available in the present dataset, however future research should examine these risk factors alongside those investigated in the present study as a means of further guiding intervention efforts. Finally, whilst for some lifestyle factors, risk was defined with reference to national guidelines (i.e., alcohol use, sleep and BMI) for others there were no guidelines available to determine risk thresholds (i.e., drug use, unprotected sex and smoking). However, due to their illegality and/or the dire consequences associated with these behaviours, it could be argued that any involvement in these behaviours constitutes significant risk.

## Conclusions

In a large, nationally representative sample of Australian adolescents, a lifestyle risk index comprised of risky alcohol use, drug use, unprotected sex, sleep duration and smoking was a fair to good predictor of health outcomes associated with the leading fatal (suicide attempt and self-harm) and non-fatal (major depression and severe psychological distress) global burdens of disease amongst adolescents. This study indicates that this lifestyle risk index represents a useful summary metric in the context of adolescent health promotion and non-communicable disease prevention. Lifestyle risk factors were found to cluster in adolescence, providing further support for the implementation of multiple health behaviour change interventions over those with a single behaviour focus. Future research should focus on determining whether this lifestyle risk index predicts adolescent health outcomes in a longitudinal framework.

## Abbreviations

AUC: Area Under the Curve
BMI: Body Mass Index
DISC-IV: Diagnostic Interview Schedule for Children 4th Edition
K10: Kessler Psychological Distress Scale
MDD: Major Depressive Disorder
YRBSS: Youth Risk Behavior Surveillance System
